# Acute Effects of 2S-Hesperidin Supplementation on Performance and External Load in Female Basketball Players

**DOI:** 10.3390/sports14090408

**Published:** 2026-09-15

**Authors:** Enrique Flórez-Gil, Patricio Pérez-Armijo, Alejandro Vaquera, Alejandro Rodríguez-Fernández

**Affiliations:** 1 VALFIS Research Group, Department of Physical Education and Sports, Instituto de Biomedicina (IBIOMED), Instituto de Investigación Biosanitaria de León (IBIOLEÓN), Universidad de León, 24071 León, Spain; eflog@unileon.es (E.F.-G.); a.vaquera@unileon.es (A.V.); alrof@unileon.es (A.R.-F.); 2 Faculty of Health Sciences, Universidad Isabel I, 09003 Burgos, Spain; 3 School of Sport and Exercise Science, University of Worcester, Worcester WR2 6AJ, UK

**Keywords:** 2S-hesperidin, ergogenic aids, basketball, female athletes, performance, external load

## Abstract

The use of ergogenic aids, particularly polyphenols such as 2S-hesperidin, has gained attention due to their potential to enhance exercise performance, although evidence in team sports and female athletes remains limited. This study aimed to investigate the acute effects of 2S-hesperidin (Cardiose^®^) supplementation on physical performance and external and internal load during basketball-specific activity in female basketball players. A randomized, cross-over, single-blind design was employed in twelve competitive female players (age: 21.4 ± 2.2 years; Tier 3–4) who ingested 500 mg of 2S-hesperidin or placebo five hours before standardized testing and on-court practice. Physical performance was assessed through handgrip strength, countermovement jump (CMJ), drop jump (DJ), sprint, agility, and the 30–15 intermittent fitness test (30–15 IFT), while external and internal load variables were monitored during a standardized on-court practice designed to reflect the players’ typical training routine. In these exploratory analyses, differences favoring the 2S-hesperidin condition were observed for dominant handgrip strength, CMJ height, DJ height in the dominant leg, and end speed in 30–15 IFT , whereas no clear differences were observed in sprint, agility, or reactive strength index. External load variables showed no clear differences between conditions. Mean heart rate was lower in the supplementation condition, although this isolated finding should be interpreted cautiously in view of the small sample and the number of outcomes examined. Overall, the observed pattern suggests that acute 2S-hesperidin supplementation may be associated with selected neuromuscular and intermittent performance responses, without clear changes in external load. Given the small sample size and multiplicity of outcomes, these findings should be regarded as exploratory and hypothesis-generating rather than as confirmatory evidence of an ergogenic effect.

## 1. Introduction

The use of ergogenic aids in sport has increased considerably in recent years, driven by a growing body of evidence suggesting their potential to enhance physical performance, delay fatigue, and improve recovery processes [1,2]. In team sports such as basketball, the most commonly used ergogenic aids include caffeine [3,4], creatine [5], beta-alanine [6], and nitrate-rich [7] supplements, which have been reported to influence variables such as repeated sprint ability, performance, and fatigue resistance [8,9,10].

Among ergogenic aids, polyphenols have attracted particular attention due to their antioxidant properties and their potential role in modulating physiological responses to exercise [11]. Hesperidin, a citrus-derived flavonoid, has emerged as a compound of interest within this group. Specifically, its naturally predominant form, 2S-hesperidin (Cardiose^®^), has been proposed to influence endothelial function, nitric oxide bioavailability, and mitochondrial efficiency [12]. These mechanisms could theoretically contribute to improvements in exercise performance; however, evidence in team-sport athletes remains limited and inconsistent, and the extent to which such mechanisms translate into measurable changes is not well established [13]. In addition to these physiological mechanisms, polyphenols such as hesperidin may influence attenuating exercise-induced oxidative stress and modulating vascular responses during high-intensity efforts [14,15]. This may be relevant in intermittent sports, where repeated bouts of intense activity can lead to the accumulation of fatigue-related metabolites and transient reductions in muscle efficiency [16]. Reported physiological effects have included increases in blood flow, reductions in oxidative stress markers, and improvements in metabolic efficiency, although these findings are not consistent across studies and it remains uncertain whether such responses translate into meaningful performance changes, particularly in intermittent, team-sport settings [17,18].

Previous studies have reported potential benefits of hesperidin and other polyphenols on exercise performance, particularly in variables related to power output, speed, and fatigue resistance [14,18,19]. For instance, supplementation with polyphenols has been associated with changes in time to exhaustion, peak power output, and recovery kinetics [16]. However, most of this evidence has been obtained in controlled laboratory settings or endurance-based protocols, with limited research exploring its effects in sport-specific contexts involving repeated high-intensity efforts. This represents a relevant gap, particularly for intermittent sports where performance depends on the ability to sustain repeated explosive actions.

Basketball is an intermittent team sport in which players continuously alternate high-intensity actions, such as accelerations, decelerations, changes in direction, and jumps, with brief periods of recovery [20]. These demands require not only high neuromuscular and metabolic capacities but also the ability to sustain performance across repeated explosive efforts [21]. In this context, performance is closely linked to the external load achieved during play, which reflects the physical work performed by the athlete and can be quantified through variables such as accelerations, decelerations, changes in direction, and Player Load [22,23]. Fatigue in basketball is associated with metabolic disturbances, reduced oxygen delivery, and impaired muscle efficiency, which may compromise the ability to sustain these external load demands. Therefore, strategies targeting vascular function, oxygen availability, and metabolic efficiency, such as 2S-hesperidin supplementation, could be relevant; however, their effectiveness in maintaining performance during repeated high-intensity actions in team-sport contexts remains unclear.

Although there are no studies directly examining the effects of hesperidin in basketball, evidence from other ergogenic aids suggests that improving blood flow and metabolic efficiency can enhance repeated sprint ability and reduce fatigue in team sports [24]. Given its proposed effects on nitric oxide production and mitochondrial function, 2S-hesperidin supplementation could potentially influence performance during repeated high-intensity actions, although the magnitude and consistency of such effects in applied team-sport settings remain uncertain, potentially contributing to the maintenance of external load variables such as accelerations, decelerations, and Player Load.

Despite the growing interest in nutritional strategies to optimize performance in team sports, the potential ergogenic effects of 2S-hesperidin have not been extensively investigated in basketball, particularly in female players. Moreover, it remains unclear whether acute supplementation can translate into meaningful improvements in sport-specific performance and how it may influence the external load achieved during training.

Therefore, the aim of the present study was to investigate the effects of acute 2S-hesperidin (Cardiose^®^) supplementation on physical performance and internal and external load during a standardized basketball practice designed to replicate a typical team practice in female basketball players. It was hypothesized that acute 2S-hesperidin supplementation might influence physical performance, potentially through mechanisms related to vascular function and oxygen delivery, while maintaining a similar external load profile, indicating greater efficiency during high-intensity intermittent exercise [18].

## 2. Materials and Methods

### 2.1. Experimental Design

**Figure 1:**
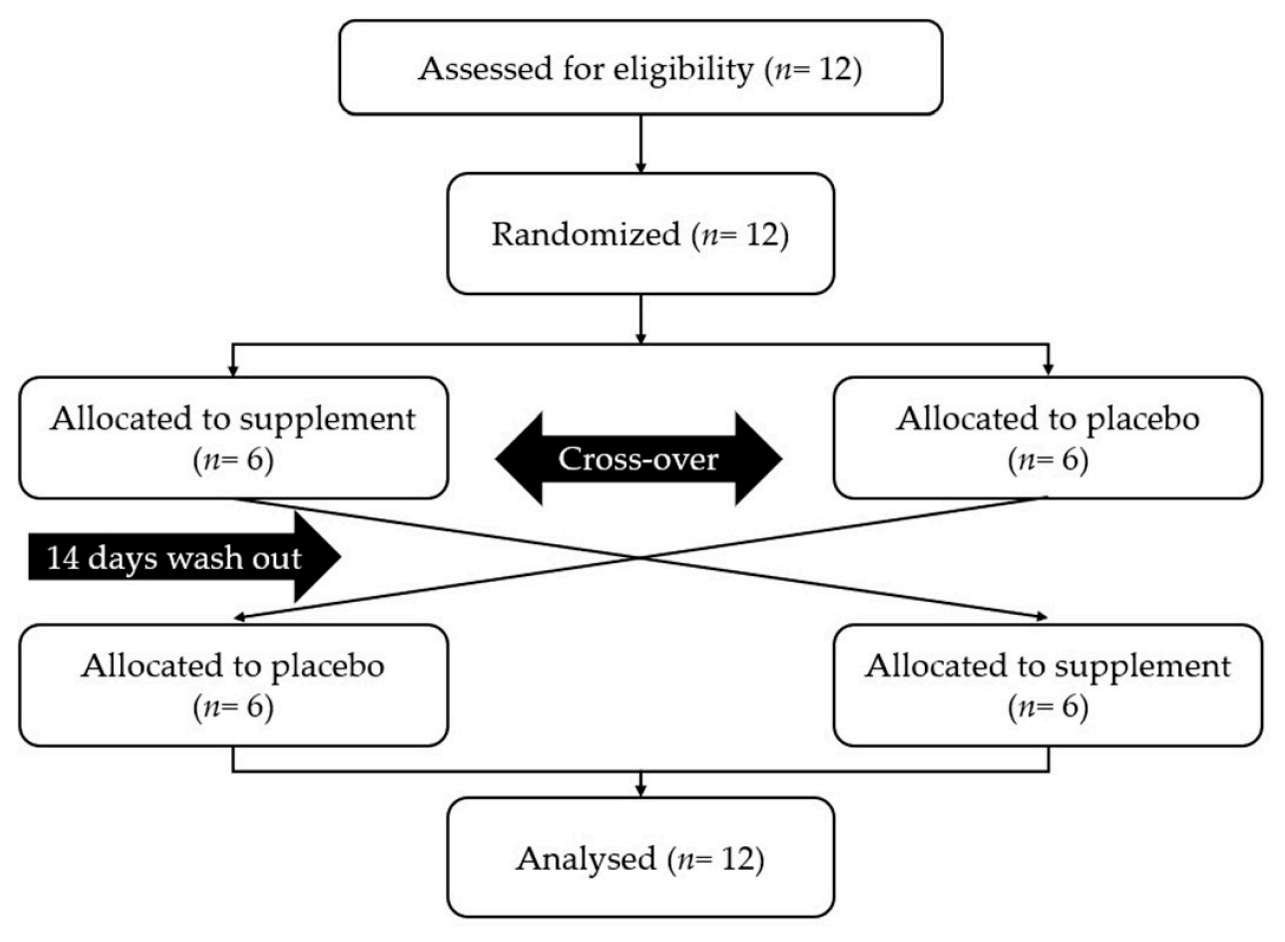
Consolidated Standards of Reporting Trials flow chart of participants during the study intervention.

A randomized, cross-over, single-blind design was used (Figure 1). The order of treatments (Cardiose^®^ vs. Placebo) was randomized using a computer-generated random sequence. A washout period of 14 days was implemented between conditions to minimize carry-over effects. The study was conducted in a single-blind manner, where participants were unaware of the treatment conditions, while researchers responsible for data collection were aware of the allocation. Both the supplement and placebo were provided in identical capsules and were indistinguishable in appearance, taste, and smell. Participants completed a total of two assessment sessions, ingesting either Cardiose^®^ (500 mg containing 90% hesperidin, of which at least 85% corresponds to the 2S-hesperidin isomer, derived from sweet orange [*Citrus sinensis*]) or a placebo (500 mg of microcrystalline cellulose) five hours before each session; both products were supplied by HealthTech BioActives (Murcia, Spain). The five-hour pre-test ingestion timing was selected based on the pharmacokinetic profile of hesperidin, as previous studies have reported peak plasma concentrations of its metabolites approximately 5 h after ingestion [18]. The study was conducted over four weeks during the middle of the competitive season. All assessments were carried out on the same day of the week (Wednesday), between 18:50 and 20:00 h, coinciding with the team’s regular gym-based training session, and were always performed on the training court. Testing sessions were scheduled for the third day after the match (MD + 3), thus ensuring a minimum of 72 h of recovery to ensure a consistent recovery status and minimize fatigue-related variability. Throughout the study, players were instructed to maintain appropriate pre-testing habits, including adequate sleep, proper hydration, and a balanced diet, and these instructions were reiterated verbally before each testing session. Also, participants recorded their dietary intake during the 24 h prior to the first session and were instructed to replicate it before the second session. Compliance with pre-testing instructions was self-reported, and participants were reminded verbally before each session to ensure consistency.

Players completed the following battery of physical fitness tests: handgrip strength (HG), countermovement jump (CMJ), single-leg drop jump (DJ), a 20 m sprint test, a modified agility test (MAT), and the 30–15 intermittent fitness test (30–15 IFT). Immediately after completing the fitness tests, players were fitted with heart rate monitors and local positioning system (LPS) devices to record the subsequent training session; session content was standardized across all recording sessions. Players were already familiar with the use of heart rate monitors and LPS devices, as well as with all fitness tests, since these were routinely incorporated into the team’s regular training and monitoring procedures. All players completed all tests and the subsequent training sessions on the corresponding testing days. All tests were conducted by the same researchers using standardized procedures to ensure consistency across sessions.

### 2.2. Subjects

Twelve female basketball players (mean ± SD; age: 21.4 ± 2.2 years; height: 177 ± 9.3 cm; body mass: 74.9 ± 11.6 kg) from the same club participated in the study. The players had competed in the Spanish third division for four consecutive seasons, achieving promotion to the second-highest category of Spanish basketball, and were classified as Tier 3/4 according to the Subject Classification Framework [25]. All participants had a minimum of five years of competitive basketball experience and regularly engaged in four weekly training sessions lasting between 45 and 100 min, in addition to playing an official national league match each weekend. One session consisted of gym-based resistance training combined with coordination and running technique exercises (approximately 45 min), whereas the remaining three sessions were team-based on-court practices (approximately 100 min), primarily focused on game-based drills. The club, coaching staff, and players were informed about the study procedures, as well as the potential risks and benefits, prior to the beginning of the study. All participants provided written informed consent. The study protocol was approved by the Ethics Committee of the Universidad de León (ETICA-ULE-067-2024) and adhered to the principles outlined in the Declaration of Helsinki.

### 2.3. Procedures

#### 2.3.1. Physical Fitness Testing

Before testing, participants performed a standardized warm-up that consisted of 10–15 min of low-intensity running, dynamic mobility exercises, and progressive neuromuscular activation exercises. This protocol was designed following recommendations from the scientific literature regarding basketball players to ensure consistent physiological preparation and reduce the risk of injuries in all test sessions [26]. After the warm-up, the participants performed the physical performance tests in a fixed order under the supervision of experienced researchers. A standardized rest period of 3 min was provided between tests.

The dominant limb was determined based on self-report (preferred hand/leg used for sports-specific actions).

Isometric upper-body strength was assessed using a digital handgrip dynamometer (Takei TKK-5401, Takei Scientific Instruments, Niigata, Japan), a valid and reliable indicator of overall neuromuscular function [27]. Participants performed two maximal attempts with each hand in an alternating order, following a standardized standing protocol. The arm was fully extended alongside the body without contacting the thigh, exerting maximal force for 5 s. A 45 s rest period was allowed between attempts with the same hand, and the highest value recorded (kg) was used for analysis.

Lower-body explosive performance was evaluated through CMJ and unilateral DJ tests. For the CMJ, participants started from an upright standing position, performed a rapid downward movement to approximately 90° of knee flexion, and executed a maximal vertical jump while keeping their hands on their hips throughout the movement. Two attempts were performed with 2 min of recovery between trials, and the highest jump height (cm) was retained. For the unilateral DJ, participants stepped off a 30 cm box, landing on one leg and immediately performing a maximal vertical jump with the same leg, without any upward propulsion during the drop phase. Hands remained on the hips at all times. Jump height (cm) and reactive strength index (RSI) [28] were measured using an infrared optical system (Optojump Next, Microgate, Bolzano, Italy), with RSI automatically calculated as the ratio between jump height (m) and ground contact time (s).

Linear sprint performance was assessed over 20 m. Participants performed two maximal sprints with 5 min of recovery between attempts, and the fastest time was retained. Sprint times were recorded using wireless photocell timing gates (Witty, Microgate, Bolzano, Italy). The first gate was positioned 0.5 m in front of the starting line to avoid premature triggering, with subsequent gates placed at 5 m and 20 m. Each gate consisted of a transmitter and receiver unit separated by 3 m. Timing was automatically triggered when participants crossed the first gate.

Change-of-direction ability was evaluated using the MAT, which reflects sport-specific movement patterns in basketball. The protocol required participants to sprint forward, perform lateral displacements, and backpedal to the starting line. Two trials were completed with 2 min of recovery, and the fastest time was recorded using a photocell system (Witty, Microgate, Bolzano, Italy) positioned at the start and the finish line.

Intermittent aerobic fitness and maximal intermittent running velocity (VIFT) were determined using the 30–15 IFT, following a basketball-specific adaptation [29]. The test consisted of repeated 30 s shuttle runs over 28 m interspersed with 15 s passive recovery periods. Initial speed was set at 10 km·h^−1^ and increased by 0.5 km·h^−1^ at each stage. Running pace was dictated by auditory signals emitted from a pre-recorded audio file, and participants were required to adjust their running speed to reach the designated zone in synchrony with the beeps. The audio signals were delivered through a validated mobile application. The test ended when participants failed to reach the required zone on three consecutive occasions or upon volitional exhaustion. The final completed speed was recorded.

#### 2.3.2. Training Session and Load Monitoring

After completing the testing battery, participants remained seated during the 30 min passive recovery period and had access to water ad libitum before performing the training session. This recovery period was included to minimize the potential influence of fatigue induced by the testing procedures, while maintaining consistency across all experimental sessions. Following this recovery period, participants completed a standardized 90 min on-court training session in which both internal and external load were recorded. This approach was used to replicate real-world training conditions, and the order of procedures was kept consistent across all experimental sessions to minimize potential bias derived from accumulated fatigue.

Practice content and structure were standardized across all sessions to ensure comparable load demands. Each session included an initial activation phase with low-intensity running, mobility, and dynamic movements, followed by individual technical drills focused on ball handling, passing, and shooting drills. Subsequently, players performed technical-tactical tasks involving structured offensive and defensive situations. The main part of the session consisted of small-sided games (SSGs) under different formats designed to replicate game demands. Finally, the session concluded with full-court 5 vs. 5 play to simulate real match conditions.

#### 2.3.3. Internal Load

Internal load was assessed through heart rate (HR) responses and session rating of perceived exertion (sRPE). HR was continuously recorded using chest-worn bands (HRM-Dual; Garmin Ltd.; Olathe, KS, USA), which transmitted data to the WIMU PRO system (RealTrack Systems, Almería, Spain) with a sampling frequency of 4 Hz. Peak (HRpeak) and mean (HRmean) HR responses were determined during each practice. This device was previously validated with strong correlations reported with the Polar Team 2 system (r = 0.958, *p* < 0.001) [30]. Data were analyzed using the system-specific software (S PRO version 1.0.0; RealTrack Systems).

In addition, sRPE was collected individually 10 min after the completion of each training session using the Borg CR-10 scale [31].

#### 2.3.4. External Load

External load was monitored using a local positioning system based on ultra-wideband technology and equipped with triaxial accelerometers (WIMU PRO™, RealTrack Systems, Almería, Spain). Positional data were sampled at 100 Hz and processed using dedicated software (SPRO™, version 1.0.0; RealTrack Systems, Almería, Spain). Each player wore the same device across all sessions to minimize interunit variability. Devices (85 × 48 × 15 mm; 65 g) were placed in a manufacturer-provided vest positioned on the upper back and fitted approximately 15 min before the warm-up. Units were activated 30 min before each session and calibrated according to the manufacturer’s guidelines, with warm-up data excluded from analysis. The validity of this LPS is superior to global positioning system devices, with total biases from 20.56 to 0.67% for movement velocity and from 21.38 to 1.51% for movement distance during linear, curvilinear, and zig-zag team sport–based activities performed at different intensities in comparison to reference methods (infrared timing cells and trundle wheel measurements [30].

**Figure 2:**
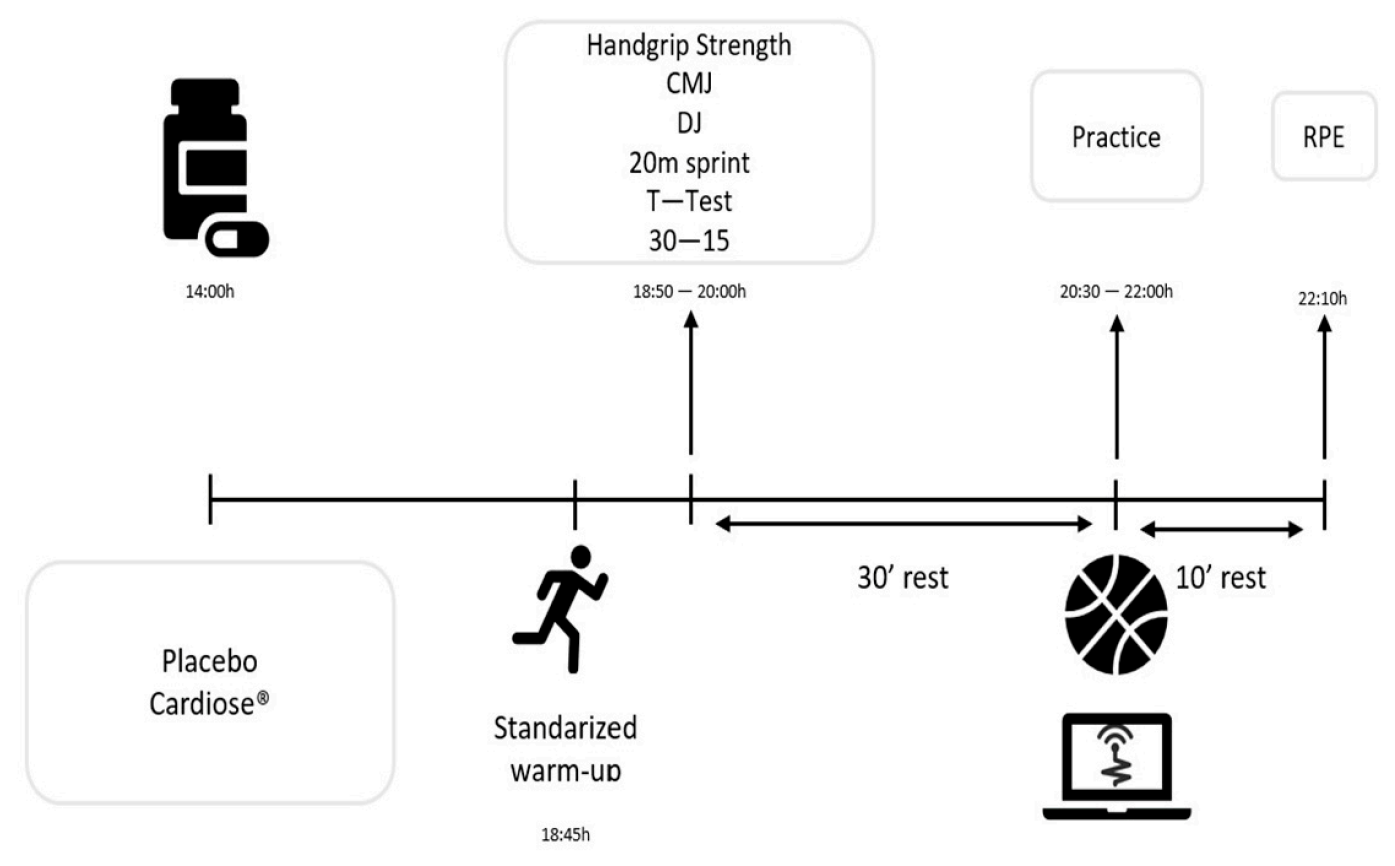
Overview of the experimental protocol showing supplementation intake, standardized warm-up, physical performance tests, on-court training session, and subsequent internal load monitoring (RPE).

The following external load variables were collected: total distance covered (m), total explosive distance (m), distance covered while accelerating and decelerating (m), maximum and average acceleration and deceleration values (m/s^2^), high-intensity accelerations and decelerations distances (m), and player load (arbitrary units). The overall structure of each experimental session is presented in Figure 2.

### 2.4. Statistical Analysis

Descriptive statistics are presented as mean ± standard deviation (SD). Normality of data distribution was assessed using the Shapiro–Wilk test, confirming that the assumption of normality was met for all variables; parametric tests were therefore used throughout. For each outcome variable, the percentage difference between conditions was calculated on an individual basis as Δ (%) = [(Cardiose^®^ value − placebo value)/placebo value] × 100. Paired-sample *t*-tests were used to compare each outcome variable and external and internal load between the two experimental conditions (Cardiose^®^ vs. placebo). Statistical significance was set at α = 0.05 [32].

To provide a more transparent account of variability, linear mixed-effects models were used for each outcome variable. In these models, condition (Cardiose^®^ vs. placebo) was included as a fixed effect and participant as a random effect (random intercept). This approach allows the decomposition of total variability into between-participant variance and residual (within-participant) variance, which were explicitly reported alongside the estimated condition effect and its 95% confidence interval. The residual variance was interpreted in relation to previously reported reliability values (coefficient of variation and intraclass correlation coefficient) to assess whether observed differences exceeded typical measurement noise. All estimates are presented with 95% confidence intervals. *p*-values are reported for completeness but were interpreted in the context of variance components and measurement variability rather than as sole indicators of effect. Statistical analyses were performed using Jamovi (version 2.4, The Jamovi Project, Sydney, Australia).

Test–retest reliability for the performance tests used in this study has been previously reported in comparable populations. Specifically, countermovement jump and drop jump tests typically show coefficients of variation (CV) of ~3–5% and high intraclass correlation coefficients (ICC > 0.90); handgrip strength presents excellent reliability (ICC > 0.95); sprint tests show CV values of ~1–3%, and the 30–15 intermittent fitness test has been reported to have a CV of ~2–3% in trained team-sport athletes [33,34,35]. These values are consistent with those observed in studies involving competitive female team-sport athletes, including basketball players with similar training status and competitive level.

Importantly, these reliability values were selected because they originate from studies involving trained or semi-professional team-sport populations, which are comparable to the present sample of Tier 3–4 female basketball players competing at national level. Therefore, they provide an appropriate reference framework for interpreting whether observed within-subject changes exceed typical measurement error in this specific athletic context.

Given the small sample size and the acute nature of the intervention, statistical analysis should be considered exploratory. Therefore, the results are interpreted with caution, focusing on the magnitude and direction of the effects rather than on dichotomous significance testing. Given the crossover design, the potential influence of period and sequence effects was considered; however, due to the limited sample size and the reduced statistical power to reliably estimate additional parameters, a linear mixed-effects model including participant as a random effect and condition, period, and sequence as fixed effects was not implemented. Instead, paired sample *t*-tests were retained as a simpler analytical approach within this exploratory framework. Consequently, potential period or carryover effects cannot be ruled out and should be considered when interpreting the findings.

## 3. Results

Physical fitness outcomes under the Cardiose^®^ and placebo conditions are presented in Table 1. Differences between conditions were estimated using linear mixed-effects models including condition as a fixed effect and participant as a random effect. In addition to the estimated condition effects and their 95% confidence intervals, between-participant and residual (within-participant) variance components are reported to provide a transparent account of variability and to allow interpretation of the observed differences relative to typical within-participant variability. Descriptive statistics are also presented to facilitate comparison between conditions.

Importantly, the four outcomes that reached statistical significance (dominant handgrip strength, CMJ, dominant-leg DJ height, and 30–15 IFT final velocity) should not be interpreted as independent confirmations of a supplementation effect. These measures are likely to be correlated within a given session, as they reflect related neuromuscular and performance capacities that may fluctuate together due to participant-specific factors (e.g., daily readiness, fatigue, or motivation). Therefore, a participant experiencing a generally “better” or “worse” session could exhibit concurrent changes across multiple outcomes, independent of the supplementation condition. Consequently, these findings should be interpreted collectively as a pattern of responses rather than as multiple independent pieces of evidence, and the overall strength of inference should be considered accordingly.

External and internal load outcomes under the Cardiose^®^ and placebo conditions are presented in Table 2. Differences between conditions were estimated using linear mixed-effects models including condition as a fixed effect and participant as a random effect. In addition to the estimated condition effects and their 95% confidence intervals, between-participant and residual (within-participant) variance components are reported to provide a transparent description of the variability structure underlying each outcome and to allow interpretation of the magnitude of the observed differences relative to typical within-subject variation. Descriptive statistics are also included to facilitate comparison between conditions.

## 4. Discussion

The aim of this study was to analyze the effect of acute 2S-hesperidin (Cardiose^®^) supplementation on physical performance and internal load in female basketball players. The main findings suggest that a single acute dose of the supplement may be associated with small improvements in selected neuromuscular and intermittent-exercise performance, with statistical differences observed in dominant handgrip strength, countermovement jump height, drop jump height on the dominant leg, and final velocity in the 30–15 intermittent fitness test. The linear mixed-effects models indicated that variability was largely driven by between-participant differences, whereas residual (within-participant) variance was comparatively lower across most outcomes, providing context for interpreting the magnitude of the observed condition effects.

Dominant handgrip strength increased by 2.8% under the Cardiose^®^ condition relative to placebo (*p* = 0.005), which may reflect a possible acute enhancement in maximal force production. This effect is particularly relevant when considered in relation to the residual variance observed in handgrip strength, which provides context for within-participant variability and suggests that the observed change is unlikely to be explained solely by measurement noise. Similarly, final velocity in the 30–15 intermittent fitness test was 4.1% higher in the supplementation condition (*p* = 0.028), indicating a potential improvement in intermittent high-intensity running capacity, a key determinant of basketball match performance. The residual variance observed in this test provides context for the magnitude of within-participant variability relative to the observed condition effect.

In contrast, no clear differences were observed in 20 m sprint time, agility performance, or reactive strength index, suggesting that any acute effect of 2S-hesperidin may be selective and task-dependent rather than uniformly affecting all neuromuscular qualities. From a variability perspective, the higher between-participant variance relative to residual variance indicates that a substantial proportion of variability is attributable to stable inter-individual differences, which should be considered when interpreting the absence of condition effects.

Regarding training load, mean heart rate was lower under the Cardiose^®^ condition despite comparable external load between conditions, pointing toward a potential reduction in cardiovascular demand for a similar external load. This difference should be interpreted in the context of the variance estimates from the mixed-effects models, which provide information on the relative contribution of between-participant and within-participant variability. However, this difference alone does not allow inference of physiological adaptation or efficiency changes.

Accordingly, any interpretation in terms of improved cardiovascular efficiency remains speculative, as the data do not allow disentangling physiological adaptation from normal session-to-session variability. Collectively, these findings suggest that acute 2S-hesperidin supplementation may have potential practical implications for explosive, dominant-limb, and intermittent-effort actions in basketball, although the magnitude and consistency of these effects should be interpreted in relation to within-participant variability and the correlated nature of performance outcomes. However, given the within-participant variability and the likely correlation between performance outcomes within a session, these findings should be considered exploratory.

From a physiological perspective, these results may be partly related to acute neuromuscular and perceptual responses to supplementation, although the exact mechanisms remain unclear due to the absence of physiological measurements [19,36,37]. Importantly, the variability structure observed in the linear mixed-effects models indicates that these responses occur within a context of substantial inter-individual differences, with between-participant variance consistently exceeding within-participant (residual) variance across outcomes, highlighting that a substantial proportion of outcome variability is attributable to stable individual differences.

Regarding upper body strength, the higher dominant handgrip values observed under the Cardiose^®^ condition suggest a possible acute improvement in maximal force-production capacity, which is consistent with previous research describing favorable effects of hesperidin on muscle function [18]. This type of improvement may have practical implications for specific game actions, such as ball control, physical contact, and stability in opposing situations [14,18,38]. In addition, the relatively low residual variance observed in handgrip strength indicates that within-subject variability was minimal, providing context for within-subject variability without implying measurement reliability conclusions, whereas the high between-participant variance highlights substantial inter-individual differences in absolute strength levels.

On the other hand, the results obtained in the jump tests showed higher jump heights in both the CMJ and the DJ in the dominant leg. These observations may indicate a possible acute effect on lower-body explosive performance following supplementation, which is consistent with previous studies that have reported improvements in power output after flavonoid interventions [18,38,39]. However, the predominance of between-participant variance over residual variance in these outcomes indicates that inter-individual variability accounts for a large proportion of observed differences, which should be considered when interpreting condition effects.

Regarding sprint performance, although 20 m sprint time was numerically faster under the Cardiose^®^ condition (Δ = −2.6%), this difference did not reach statistical significance. Therefore, this observation should be interpreted with caution rather than as a clear performance trend, suggesting that the benefits of supplementation may not clearly translate to sustained linear speed actions. Similarly, the lack of changes in the agility test indicates that 2S-hesperidin does not appear to influence complex abilities that depend on coordination, decision-making, and joint stability. From a variability perspective, the higher between-participant variance combined with low residual variance in these variables indicates that these abilities are strongly trait-dependent and relatively stable within individuals, which may partly explain the absence of detectable condition effects within the present sample.

One of the most notable observations of the study was the higher final speed in the 30–15 test, suggesting a possible positive effect on intermittent aerobic capacity. When interpreted in the context of the variance structure, the relatively low residual variance suggests good test sensitivity, whereas the high inter-individual variability indicates heterogeneous baseline aerobic capacity, which may influence the magnitude of the ergogenic response.

In terms of external load, no clear differences were observed between conditions, suggesting that supplementation did not appear to modify the amount of physical work performed during the practice. Although small directional differences were observed in specific variables (e.g., Player Load and high-intensity acceleration/deceleration distance), these changes were of low magnitude and remained within the range of within-participant (residual) variability reported for these measures (e.g., Player Load residual variance = 131.9; High Acc = 1909; High Dec = 1093), indicating limited evidence for a systematic effect of supplementation on external load distribution. Given the small effect sizes and non-significant *p*-values for these variables, this pattern should be regarded as descriptive rather than indicative of a true training-load effect, and is more consistent with normal session-to-session variability than with a condition-driven change in locomotor demands.

From the perspective of internal load, mean heart rate was lower under the Cardiose^®^ condition (*p* = 0.031), whereas peak heart rate was numerically higher (*p* = 0.078). When interpreted alongside variability estimates, HRmean showed relatively lower residual variability (34.1) compared with its between-participant variance (136.5), suggesting a more stable and sensitive response to condition, whereas HRpeak exhibited greater uncertainty (residual variance = 9.98), which may partly explain the lack of consistency between both measures. While the lower mean heart rate may suggest a potential reduction in cardiovascular demand during the session, the higher HRpeak indicates that maximal cardiovascular strain was not reduced and may even have been slightly elevated. Consequently, the internal load responses should be interpreted as reflecting heterogeneous physiological strain within sessions rather than a uniform shift in cardiovascular efficiency [40].

An additional factor that should be considered when interpreting the lower mean heart rate observed in the supplementation condition is the potential influence of the 30 min passive recovery period performed between the fitness-testing battery and the subsequent on-court practice. However, given the absence of direct physiological or recovery markers during this interval, it is not possible to determine whether supplementation influenced recovery kinetics or whether the observed differences fall within expected within-participant variability across sessions. Therefore, the observed reduction in mean heart rate may not necessarily reflect a direct effect during the practice itself, but rather a carry-over effect from an enhanced recovery state prior to its onset. Accordingly, this interpretation remains speculative, and the observed effect should be considered in light of the overall variability structure of the internal load measures rather than as evidence of a distinct recovery-mediated mechanism.

From an applied perspective, these results suggest that acute supplementation with 2S-hesperidin could be useful for improving explosive actions and intermittent capacity in basketball, potentially without increasing external workload or perceived physiological strain. This could translate into better performance in high-intensity situations during the game, while maintaining a controlled physiological cost. However, its impact appears limited on variables such as agility or maximum speed, so its use should be contextualized according to the specific demands of the sport.

Despite the study’s methodological rigor, several limitations must be considered. The relatively small sample size (*n* = 12) and the inclusion of players from only one team limit the statistical power and generalizability of the findings. Although this is common in studies involving competitive female team-sports athletes, and the participants can be classified as Tier 3–4 according to the established framework, the results should be interpreted with caution and considered preliminary, requiring confirmation in larger samples. Another important limitation is the absence of test–retest reliability data (e.g., typical error, coefficient of variation, or intraclass correlation coefficients) for the performance tests in this specific cohort, which limits the ability to determine whether the observed within-subject changes exceed normal measurement variability. Furthermore, an important limitation is the absence of biochemical or physiological markers, which prevents direct assessment of the mechanisms underlying the observed effects. In addition, the single-blind design may represent a limitation, as a double-blind approach would further reduce the risk of expectancy bias. Similarly, the lack of control over the menstrual cycle represents another significant limitation, given its potential impact on physical performance and physiological response. Furthermore, it was not possible to strictly control external variables such as sleep or stress prior to the practices.

From a practical perspective, acute 2S-hesperidin supplementation may be particularly useful in contexts where repeated high-intensity efforts are required, such as training sessions or match-play scenarios. Its use may be especially relevant when athletes aim to optimize neuromuscular performance or reduce physiological strain without altering external workload.

Finally, since the study focuses on the effects of an acute dose, it is not possible to extrapolate these results to chronic supplementation protocols. Future research should analyze the effect of prolonged interventions, explore potential synergies with other supplements, and increase the sample size to improve the external validity of the findings.

## 5. Conclusions

Acute 2S-hesperidin (Cardiose^®^) supplementation was associated with improvements in selected neuromuscular and intermittent-exercise performance in female basketball players, with no comparable effect observed for linear speed, agility, or reactive strength. This pattern may indicate that any potential ergogenic influence could be more relevant to actions involving maximal force and repeated high-intensity efforts rather than to physical fitness broadly. Regarding training load, supplementation did not appear to alter external demands but was associated with a lower cardiovascular response, which may point toward a potential acute effect on internal load management without indicating any reduction in training output.

Overall, acute 2S-hesperidin supplementation may offer a targeted ergogenic effect for explosive, dominant-limb, and intermittent-capacity actions central to basketball performance, while maintaining the players’ ability to sustain external load. Given the acute, single-dose design and the limited sample size, these conclusions should be considered preliminary and interpreted with caution; confirming their consistency across chronic supplementation protocols and larger, more heterogeneous samples remains a necessary next step before translating these findings into applied recommendations.

These improvements may translate into better performance in basketball-specific actions such as jumping, repeated high-intensity efforts, and physical contacts during play.

## Figures and Tables

**Table 1:** Physical fitness test outcomes (mean ± standard deviation) following Cardiose^®^ supplementation versus placebo in female basketball players.

	Cardiose^®^ (Mean ± SD)	Placebo (Mean ± SD)	Δ (%)	*Mean Difference (95% CI)*	*p*	Between-Participant Variance	Residual Variance
HG d (kg)	**39.6 ± 8.0**	**38.5 ± 8.2**	**2.8**	**2.12 (0.83 to 3.41)**	**0.005**	**39.1**	**2.8**
HG nd (kg)	37.8 ± 6.7	37.5 ± 7.3	0.8	1.07 (−1.66 to 3.80)	0.398	27.3	1.3
CMJ (cm)	**31.6 ± 4.7**	**28.9 ± 5.3**	**8.5**	**3.22 (0.11 to 6.33)**	**0.044**	**21.9**	**0.5**
DJ d h (cm)	**14.8 ± 2.9**	**14.0 ± 2.3**	**5.7**	**1.27 (0.07 to 2.46)**	**0.040**	**5.9**	**1.2**
DJ nd h (cm)	13.9 ± 3.0	14.0 ± 2.9	−0.7	0.00 (−0.05 to 0.04)	0.868	7.8	1.8
DJ d RSI (m/s)	0.35 ± 0.1	0.35 ± 0.2	0.0	−0.17 (−1.62 to 1.29)	0.798	0.003	0.001
DJ nd RSI (m/s)	0.38 ± 0.1	0.37 ± 0.1	2.7	0.01 (−0.04 to 0.06)	0.656	0.007	0.002
20 m sprint (s)	3.43 ± 0.1	3.52 ± 0.2	−2.6	−0.03 (−0.08 to 0.02)	0.118	0.01	0.002
MAT (s)	6.33 ± 0.2	6.29 ± 0.3	0.6	0.02 (−0.07 to 0.11)	0.589	0.02	0.007
30–15 IFT (km·h^−1^)	**19.6 ± 1.8**	**18.8 ± 2.6**	**4.1**	**1.00 (0.14 to 1.86)**	**0.028**	**4.03**	**0.59**

HG = handgrip; d = dominant; nd = non- dominant; CMJ = countermovement jump; DJ = Drop jump; RSI = reactive strength index; MAT = modified agility T-test; 30–15 IFT = final velocity in the 30–15 intermittent fitness test; Δ = percentage change between conditions; ES = effect size. Bolded *p*-value denotes a significant difference between conditions. Significance level *p* < 0.05. Mean difference is expressed as supplement minus placebo. Positive values indicate higher values in the supplement condition. Confidence intervals are presented to reflect the uncertainty around the estimated differences.

**Table 2:** Mean ± SD for external and internal load variables following Cardiose^®^ supplementation versus placebo in female basketball players, including percentage changes (Δ%), statistical significance (*p*), and effect sizes (ES).

	Cardiose^®^ (Mean ± SD)	Placebo (Mean ± SD)	Δ (%)	*Mean Difference (95% CI)*	*p*	Between-Participant Variance	Residual Variance
*External Load*							
TD (m)	4901 ± 644	4971 ± 516	−1.4	−70 (−450 to 310)	0.805	94,552	201,072
TED (m)	647 ± 144	641 ± 83.6	0.9	6 (−90 to 102)	0.890	8511	5539
Acc (m)	2177 ± 197	2233 ± 204	−2.5	−56 (−190 to 78)	0.484	24,571	12,948
Dec (m)	2180 ± 197	2241 ± 204	−2.8	−61 (−200 to 78)	0.445	23,949	13,398
Max Acc (m/s^2^)	5.32 ± 0.5	5.43 ± 0.8	−0.7	−0.11 (−0.45 to 0.23)	0.704	0.09	0.31
Max Dec (m/s^2^)	−6.12 ± 0.9	−5.72 ± 0.2	−2.7	−0.40 (−1.05 to 0.25)	0.212	0.05	0.45
AVG Acc (m/s^2^)	0.821 ± 0.1	0.816 ± 0.1	0.6	0.005 (−0.03 to 0.04)	0.780	0.005	0.001
AVG Dec (m/s^2^)	−0.818 ± 0.1	−0.808 ± 0.1	1.2	−0.010 (−0.05 to 0.03)	0.533	0.004	0.001
High Acc (m)	273 ± 99.3	255 ± 83.1	6.6	18 (−45 to 81)	0.441	7232	1909
High Dec (m)	247 ± 86.1	231 ± 56.1	6.5	16 (−40 to 72)	0.290	4681	1093
Player Load (u.a.)	69.6 ± 13.1	73.4 ± 15.1	−5.5	−3.8 (−12.5 to 4.9)	0.514	67.2	131.9
*Internal Load*							
sRPE	6.9 ± 0.2	7.0 ± 0.2	−1.4	−0.1 (−0.3 to 0.1)	0.348	0.806	0.389
Hrpeak (b·min^−1^)	185 ± 9.6	181 ± 10.2	2.1	4 (−0.5 to 8.5)	0.078	99.6	9.98
Hrmean (b·min^−1^)	**149 ± 15.** **1**	**157 ± 11.8**	**−5.4**	**−8 (−14.5 to −1.5)**	**0.031**	**136.5**	**34.1**

TD = total distance; TED = total explosive distance; Acc = accelerations; Dec = decelerations; Max Acc = maximum acceleration; Max Dec = maximum deceleration; AVG Acc = average acceleration; AVG Dec = average deceleration; High Acc = high-intensity accelerations distance; High Dec = high-intensity decelerations distance; Player Load = composite measure of external load (arbitrary units, a.u.); sRPE = Rating of perceived exertion per session; HRpeak = peak heart rate; HRmean = mean heart rate; Δ (%) = percentage change between conditions; *p* = *p*-value; ES = effect size; and bolded *p*-value denotes significant difference between conditions. Significance level *p* < 0.05. Mean difference is expressed as supplement minus placebo. Positive values indicate higher values in the supplement condition. Confidence intervals are presented to reflect the uncertainty around the estimated differences.

## Data Availability

The data presented in this study are available from the corresponding author upon reasonable request. The data are not publicly available due to privacy and ethical restrictions.

## Abbreviations

The following abbreviations are used in this manuscript:

Acc	Accelerations
AVG Acc	Average acceleration
AVG Dec	Average deceleration
CMJ	Countermovement jump
Dec	Decelerations
DJ	Drop jump
ES	Effect size
HG	Handgrip
HR	Heart rate
HRmean	Mean heart rate
HRpeak	Peak heart rate
IFT	Intermittent fitness test
LPS	Local positioning system
MAT	Modified agility T-test
Max Acc	Maximum acceleration
Max Dec	Maximum deceleration
sRPE	Rating of perceived exertion per session
RSI	Reactive strength index
SD	Standard deviation
SSG	Small-sided games
TD	Total distance
TED	Total explosive distance
VIFT	Final velocity in the 30–15 intermittent fitness test
